# Mouse spinal cord cellular mapping of dopamine D2 receptors-containing cells

**DOI:** 10.3389/fnana.2025.1724268

**Published:** 2026-01-20

**Authors:** Pauline Tarot, Laura Cutando, Laia Castell, Emma Puighermanal, Emmanuel Valjent

**Affiliations:** 1 IGF, University Montpellier, CNRS, Inserm, Montpellier, France; 2 INM, University Montpellier, Inserm, Montpellier, France

**Keywords:** dopamine D2 receptor, Ribotag methodology, smFISH, spatiomolecular diversity, spinal cord

## Abstract

The spinal cord (SC) serves as the primary relay for sensory information originating in the periphery and transmitted to the brain for processing. Sensitive primary afferent fibers project to the dorsal horn, which contains a highly diverse array of neurons forming a complex network of excitatory and inhibitory circuits. Previous studies have indicated that this neuronal network can be modulated by the monoaminergic system, particularly through the spinal dopaminergic circuit, partly via dopamine D2 receptors (D2R). However, the identity of the cells expressing D2R within the spinal cord remains largely unknown. By combining whole-mount immunostaining, volume imaging and Ribotag methodology, we analyzed the distribution and characterized the molecular identity of D2R-expressing cells of the mouse spinal cord. Our study revealed that D2R are expressed by neurons, but not glial cells, distributed preferentially in the dorsal horn of the spinal cord. Furthermore, SC D2R neurons were not motorneurons but instead belong to molecularly distinct classes of excitatory and inhibitory neuronal populations. By providing a detailed molecular characterization of D2R-expressing cells in the spinal cord, the present work lays the foundation for more targeted investigations into the specific functional roles of D2Rs in sensory information processing.

## Introduction

The spinal cord (SC) is the main relay for sensory signals from the periphery to the brain (D'Mello and Dickenson, 2008). Distinct sensory information is conveyed by primary afferent fibers that innervate dorsal horn neurons of the SC following a specific laminar organization (Kato et al., 2009; Rivera-Arconada et al., 2025; Todd, 2002). While nociceptive information carried by A-δ and C fibers is processed by neurons located in the superficial laminae (I–II), proprioceptive information conveyed by mechanosensitive A-β fibers is integrated in the deeper laminae (III–V) (Basbaum et al., 2009). Through the fine adjustment of a filtering process, molecularly distinct classes of excitatory and inhibitory neurons located in the dorsal horn contribute to the selection of relevant sensory information, ultimately optimizing action plans (Grudt and Perl, 2002; Yasaka et al., 2007; Grudt and Perl, 2002; Häring et al., 2018; Yasaka et al., 2007).

The integration of sensory information by dorsal horn neurons is also strongly modulated by descending monoaminergic pathways including diencephalospinal dopaminergic (DA) neurons arising from the A11 nucleus which influence motor and sensory functions (Koblinger et al., 2014; Ozawa et al., 2017; Qu et al., 2006). At the SC level, DA mediates its effect through the activation of D1R- (D1R and D5R) and D2R-like (D2R, D3R and D4R) receptors, as mapped by *in situ* hybridization and receptor autoradiography (Gladwell et al., 1999; Levant and McCarson, 2001; Schotland et al., 1995; Sharples et al., 2014; Van Dijken et al., 1996; Venugopalan et al., 2006; Xie et al., 1998; Zhao et al., 2007; Zhu et al., 2008). Among them, D2R is the most abundantly expressed (Sharples et al., 2014; Zhu et al., 2008) and accumulating evidence suggests that it plays a prominent role in regulating dorsal horn neuronal activity and antinociception (Dai et al., 2016; Tang et al., 2021; Van Dijken et al., 1996). However, significant gaps remain in our understanding of the specific role of D2R signaling across the different classes of dorsal horn neurons.

Here, we combined three-dimensional imaging, single molecule *in situ* hybridization, cell type-specific transcriptomics and imaging analyses to decode the identity of mouse SC D2R cells. We found that SC D2R neurons are preferentially localized in the dorsal horn where they segregate into molecularly distinct classes of excitatory and inhibitory neurons. The spatiomolecular characterization of SC D2R neurons provides new anatomical insights that may be certainly helpful to clarify the cellular mechanisms underlying D2R-mediated antinociception.

## Materials and methods

### Animals

Male and female C57BL/6 (*n* = 12) from Charles River Laboratories, *Drd2-Cre* (*n* = 4) and *Drd2-Cre:Ribotag* (*n* = 30) mice were used in the present study. All experiments were performed on mice aged 8–12 weeks. Mice were housed in groups of 2–5 per cage (standard sizes in accordance with European animal welfare guidelines 2010/63/EU) and maintained in a 12 h light/dark cycle (lights on from 7:00 a.m. to 7:00 p.m.), with stable conditions of temperature (22°C) and humidity (60%), with food and water provided *ad libitum*. All animal procedures were conducted in accordance with the guidelines of the French Agriculture and Forestry Ministry for handling animals (authorization number/license B34-172-41) and approved by the relevant local and national ethics committees (authorizations APAFIS#14880).

### Ribotag methodology

This methodology was carried out on mouse line *Drd2-Cre:Ribotag*. These mice expressed the Rpl22 ribosomal subunit tagged with hemagglutinin (HA) in D2R-expressing cells (Puighermanal et al., 2015).

### Intraspinal injection

*Drd2*-Cre mice were anesthetized with a cocktail of ketamine (10 mg/ml) and xylazine (1 mg/ml). Mice received 100–200 μl of saline solution (0.9% NaCl, s.c) and ophthalmic cream (Allergan) on their eyes to prevent dehydration. The back was shaved and disinfected using Vetadine, and a 2 cm incision was made along the spinal axis. Muscles were carefully removed with a scalpel. Mice were then placed on a stereotaxic apparatus (World Precision Instrument WPI) using a spinal adaptor (WPI) to raise the lumbar region, allowing access to the spinal cord without laminectomy. Connective tissues and muscles around the target zone (L2–L4) were gently removed until the spinal cord was visible. A glass capillary (8–10 μm tip diameter) mounted on a Hamilton syringe (65 RN, 5 μl) and fitted to an injector (UMP3 UltraMicroPump, WPI) connected to 3 axis digital controller (WPI) was used for injections. Bilateral injections of AAV9-hEF1a-DIO-XFP-InvCheTF (1.93 × 10^13^) were performed at a medio-lateral distance of 350 μm and 400 μm from the central vessel of the spinal cord and at a dorso-ventral depth of−350 μm. Viral vector (500 nl per site) was delivered into the dorsal horn of the lumbar spinal cord at a rate of 100 nl/min. After a 5 min post-injection pause, the glass capillary was slowly removed (300 μm/30 s). Incisions were closed with Coated-Vicryl 6-0 sutures (Ethicon). Mice were returned to their home cages on a heated pad until fully awake and were allowed to recover for 3 weeks.

### Tissues collection and preparation

*For immunofluorescence*. Mice were rapidly anesthetized by Euthasol administration (340 mg/kg, i.p) before intracardiac perfusion with 4% PFA (4°C). Spinal cords were extracted and post-fixed overnight at 4°C in 4% PFA. After one wash of PBS 1X, tissue was embedded in 4% agarose and sagittal slices of 40 μm were prepared using vibratome (Leica VT-1000S). Sections were stored in cryoprotectant solution (30% Ethylene glycol, 30% Glycerol, 10% Tris Buffer) at −20°C until use.

*For RNA extraction and polyribosome immunoprecipitation*. After decapitation, *Drd2-Cre:Ribotag* mice bodies were immersed in liquid nitrogen for 4 s. Spines were isolated on ice and lumbar segments were rapidly extracted. Three lumbar sections were pooled and homogenized with a glass pestle in 1 ml polysome buffer (50 mM Tris pH 7.4, 100 mM KCl, 12 mM MgCl_2_, 1% NP-40,1 mM DTT, 1 mg/ml heparin, 100 μg/ml cycloheximide, 200 U/ml RNAseOUT and protease inhibitor cocktail). Sample were then centrifuged at 1,000 × *g* at 4°C for 10 min and supernatants were collected.

*For iDISCO*. This methodology was performed on *Drd2-Cre:Ribotag* mice. Spinal cord extraction was similar to the protocol described for immunofluorescence collected tissues. Then, spinal cords were post-fixed at room temperature for 3 h and stored at 4°C in PBS 1X until using.

*For RNAscope*. After extraction, fixed spinal cords were post-fixed at room temperature during 4 h, then washed with PBS 1X. Spinal cords were then cryoconserved by immersion in a bath of increasing concentration of sucrose (10% for 4 h, 2 0% for 6 h and 30% for 24 h) under agitation at 4°C. Finally, spinal cords are embedded in OCT (Optimal Cutting Temperature) and rapidly immerged in isopentane cooled by liquid nitrogen before storage at −80°C.

### Immunofluorescence

Immunofluorescence was performed as previously described (Biever et al., 2015). Free floating sections of lumbar spinal cord were washed three times at room temperature during 10min in Tris-buffer Saline (TBS 1X) (250 mM Tris-HCL, 150 mM NaCl, pH 7.5). Permeabilization was performed by incubating the slices 20 min in 0.2% (vol/vol) Triton X-100 in TBS. Slices were then rinsed in TBS for 10 min and incubated in blocking solution (3% BSA in TBS) for 1 h at room temperature. Finally, slices were incubated for 72 h at 4°C, under checking, with primary antibodies (Table 1) previously prepared in primary antibody solution (1% BSA and 0.15% Triton X-100 in TBS). Slices were washed three times for 10 min each in TBS and incubated for 1 h with secondary antibodies (Table 2). After two washes of 10 min with TBS and one wash with Tris-buffer (0.25 M, pH 7.5), slices were mounted in DPX (Sigma-Aldrich) and stored at 4°C.

**Table 1:** List of primary antibodies.

**Antibodies**	**Species**	**Dilution**	**Supplier**	**References**
HA	Mouse	1:1,000	Biolegend	#clone 16B12
HA	Rabbit	1:500	Rockland	#600-401-384
Parvalbumin	Mouse	1:500	Millipore	#MAB1572
Parvalbumin	Rabbit	1:1,000	Swant	#PV 25
Calbindin-D28k	Rabbit	1:1,000	Swant	#CB382
Calretinin	Mouse	1:500	Swant	#6B3
Calretinin	Rabbit	1:1,000	Swant	#7699/3H
GFP	Chicken	1:1,000	Invitrogen	#A10262
RFP	Rabbit	1:1,000	MBL	#PM005
ChAT	Rabbit	1:1000	Millipore	#AB143
NeuN	Mouse	1:500	Millipore	#MAB377
IBA1	Rabbit	1:500	Wako	#019-19741
GFAP	Rabbit	1:1,000	Dako	#N1506
TH	Rabbit	1:500	Millipore	#AB152
PKCγ	Guinea Pig	1:1,000	Frontier Institute	#GP-Af350

**Table 2:** List of secondary antibodies.

**Antibodies**	**Species**	**Dilution**	**Supplier**	**References**
Anti-mouse A488	Goat	1:500	Invitrogen	A11001
Anti-Rabbit CY3	Goat	1:500	Jackson immunoresearch	115-165-075
Anti-Goat CY3	Goat	1:500	Invitrogen	A10520
Anti-Rabbit A488	Goat	1:500	Invitrogen	A11034
Anti-Guinea Pig A647	Goat	1:500	Invitrogen	A21450
Anti-Chicken A488	Goat	1:500	Invitrogen	A11039

## iDISCO tissue clearing procedure

The iDISCO tissue-clearing procedure was performed following a solvent-based protocol adapted from Renier et al. (2014) and Vigouroux et al. (2017), with minor modifications. Spinal cords were first dehydrated at room temperature through successive methanol baths (50%, 80%, and 100%) for 90 min each, then incubated overnight in methanol containing 6% hydrogen peroxide (H_2_O_2_) for tissue bleaching. Rehydration was carried out through descending methanol concentrations (100%, 80%, 50%) for 90 min per step. Samples were subsequently permeabilized and blocked for four days at room temperature in PBSG-T buffer (PBS containing 0.2% gelatin and 0.5% Triton X-100). Primary antibody incubation of anti-HA (see Table 1) was performed at 37°C under constant agitation (70 rpm) for two weeks in PBSG-T supplemented with 0.1% saponin. Next, spinal cords were washed six times (24 h each) in PBSG-T at room temperature and then incubated for two days at 37°C in PBSG-T containing 0.1% saponin and the secondary antibody (goat anti-mouse Alexa Fluor 488). After six additional washes in PBSG-T, samples were stored at 4°C until clearing. For clearing, tissues were incubated with gentle agitation (12 rpm) in 15 ml benzyl-ether-resistant Falcon tubes in the following sequence: 50% tetrahydrofuran (THF) overnight, 80% THF for 1–2 h, 100% THF for 1–2 h (twice), 100% dichloromethane (DCM) for 30 min, and finally 100% dibenzyl ether (DBE) for several hours until complete clearing was achieved. Cleared samples were stored in DBE at room temperature, protected from light.

### Single-molecule fluorescence *in situ* hybridization (RNAscope method)

RNAscope fluorescent *in situ* hybridization was performed on lumbar spinal cord sections from wild-type mice to assess the spatial distribution of target RNAs. Spinal cords were rapidly dissected following decapitation, frozen on dry ice, stored at −80°C, and sectioned at 13 μm on a cryostat at −17°C before being mounted onto Superfrost™ Ultra Plus slides. The RNAscope Fluorescent Multiplex Assay (ACD Bio, Cat. 320850) was conducted according to the manufacturer's instructions with minor adaptations, using the probes listed in Table 3. On Day 1, slides were incubated at 37°C for 1 h, washed in PBS, treated with hydrogen peroxide for 15 min, rinsed, subjected to target retrieval for 5 min, dehydrated in ethanol, and air-dried. A hydrophobic barrier was drawn around the sections, which were then incubated with Protease III for 30 min at 40°C before hybridized with the target probes for 2 h at 40°C. After two washes in RNAscope Wash Buffer, slides were stored overnight in 5 × SSC. On Day 2, signal amplification was performed with AMP1-FLv2, AMP2-FLv2, and AMP3-FLv2 (30 min each, 40°C), followed by sequential HRP-mediated detection for each fluorescence channel using HRP-C1, C2, and C3, each coupled to the corresponding fluorophore (1:1,000 in TSA buffer) and followed by HRP blocking. Sections were counterstained with DAPI, mounted with ProLong™ Gold Antifade Mountant, and stored at 4°C in the dark until imaging.

**Table 3:** List of probes RNAscope.

**Probes (gene)**	**Probes (protein)**	**Supplier**	**References**
*Drd2*	D2R	ACDBio	406501-C3
*Slc32a1*	VGAT	ACDBio	319191-C2
*Slc17a6*	VGLUT2	ACDBio	409741-C1
*Slc17a7*	VGLUT1	ACDBio	416631-C1
*Penk*	ENKEPHALIN	ACDBio	318761-C1
*Slc6a5*	GLYT2	ACDBio	409741-C1
*Tac1*	SUBSTANCE P	ACDBio	410351-C1

### Polyribosome immunoprecipitation and RNA extraction

HA-tagged-ribosome immunoprecipitation was performed in the SC of *Drd2-Cre:Ribotag* mice, as described previously (Puighermanal et al., 2017). From the supernatant obtained after spinal cord extraction (see “Tissue Collection and Preparation for RNA Extraction” section), 100 μl were transferred to a new tube and stored at−80°C as the “input” fraction. To generate the “pellet” fraction, 5 μl of HA antibody were added to the remaining supernatant (~800 μl) of each sample, followed by overnight incubation at 4°C under agitation. A second overnight incubation was then performed with magnetic beads (Invitrogen, 100.04D) under the same conditions. On the third day, beads were washed twice for 10 min each on a magnetic rack using a high-salt buffer (50 mM Tris, pH 7.4, 300 mM KCl, 12 mM MgCl_2_, 1% NP-40, 1 mM DTT and 100 μg/ml cycloheximide). RNA extraction was then performed following the manufacturer's protocol, using the RNeasy Micro Kit (Qiagen, 74004) for ribosome-mRNA complexes corresponding to the pellet fraction, and the RNAeasy Mini Kit (Qiagen, 74104) for the input fraction. RNA quality and quantity was measured using the Nanodrop 1000 spectrophotometer.

### cDNA synthesis and quantitative real-time PCR

cDNA synthesis was performed after RNA extraction from both the pellet (immunoprecipitated ribosomes) and the input fraction (10% of the homogenate supernatant) of lumbar spinal cords from *Drd2-Cre:Ribotag* mice, following previously described procedures (Cutando et al., 2021; Puighermanal et al., 2020). cDNA was generated using the SuperScript VILO cDNA Synthesis Kit (Invitrogen) with a single-cycle program consisting of 10 min at 25°C, 60 min at 42°C, 5 min at 25°C, and a final hold at 4°C. For quantitative real-time PCR, the resulting cDNA was diluted to a final concentration of 0.3 ng/μl and used as template in reactions performed with SYBR Green PCR Master Mix on a LightCycler 480 Real-Time PCR System (Roche). In Figures 2h, 4c, 5c, f, 6c, 8b, and Supplementary Figure 2, mRNA levels in the immunoprecipitated fraction (pellet) were compared with those in the input fraction. For each gene, values were normalized to the housekeeping gene *Gapdh* and expressed relative to the input, which was set to 1. Primer sequences are listed in Table 4. Fold change was calculated using the ΔΔCt method. A total of five to nine biological replicates, each consisting of pooled tissue from three samples, were analyzed in these experiments.

**Table 4:** List of primers.

**Genes**	**Proteins**	**Primers sequences**
*β-actin*	β-Actin	F: CCCCCAGCCAAGAAAGCTAT
		R: GCCCCACCGTGTGACATC
*Gapdh*	GAPDH	F: CAACTACATGGTCTACATGTTCCAA
		R: CCCATTCTCGGCCTTCACT
*Slc32a1*	VGAT	F: TCACGACAAACCCAAGATCAC
		R: GTCTTCGTTCTCCTCGTACAG
*Gad1*	GAD	F: TTGTGCTTTGCTGTGTTTTAGAGA
		R: CCCCCTGCCCAAAGATAGAC
*Slc18a3*	VACHT	F: GGCCTCGCTACCCCACAGAA
		R: CCCAGGCCAATAAGCAGCGG
*Slc17a6*	VGLUT2	F: CGCTGTCGGGGATGGTTTGC
		R: GTGGACGAGTGCAGCAATGAGG
*Penk*	ENKEPHALIN	F: CTACAGTGCAGGCGGAATGC
		R: GTCCTTCACATTCCAGTGTGC
*Chat*	CHAT	F: CCATTGTGAAGCGGTTTGGG
		R: GCCAGGCGGTTGTTTAGATACA
*Drd2*	D2R	F: CTCTTTGGACTCAACAACACAGA
		R: AAGGGCACGTAGAACGAGAC
*Cnp*	CNP	F: GCTGCACTGTACAACCAAATTCTG
		R: ACCTCCTGCTGGGCGTATT
*Gfap*	GFAP	F: AGCGAGCGTGCAGAGATGA
		R: AGGAAGCGGACCTTCTCGAT
*Aif1*	IBA1	F: CCCCCAGCCAAGAAAGCTAT
		R: GCCCCACCGTGTGACATC
*Itgam*	CD11b	F: ATGGTCACCTCCTGCTTGTGAG
		R: CCAGCAGTGATGAGAGCCAAGA
*S100b*	S100B	F: TTCCACCAGTACTCCGGGCG
		R: GCGACGAAGGCCATGAACTCC
*Tlx3*	TLX3	F: AAATGACGGACGCGCAGGTC
		R: GAAGGCGTCGTGTTGCAGCT
*Pax2*	PAX2	F: CCATCCCCAGTACACCGCCT
		R: TCCCCGCGGTAACTAGTGGC
*Tac1*	SUBSTANCE P	F: TGGACTAATGGGCAAAAGAGC
		R: CGTTCACTGCTCACTGACAC
*Slc17a7*	VGLUT1	F: TGTGCCCCATCATCGTGGGT
		R: CGCTCATCTCCTCCGGCTCT
*Slc6a5*	GLYT2	F: TGATGCTCGCCTGCTCCGTT
		R: CCCGCGGTGCTGAGCTAAGA

## Imaging and quantification

Images were taken by using confocal microscope Zeiss LSM-780. 10X and 20X objectives were used for lumbar spinal cord sections. For image acquisition, the following imaging steps were performed: z-stack and mosaic for whole coronal slice of spinal cord and snaps with 10X for one hemisphere of coronal slice. Images were quantified using ImageJ. Coronal section of half spinal cord was manually organized by laminae (I–X). An average of 4 slices of the same animal was counted. For the calculation of the quantification percentage, we decided to represent the colocalization (1) relative to HA- or *Drd2*-positive neurons (HA^+^/*Drd*2^+^) neurons and (2) relative to the marker of interest (e.g., *Slc17a6, Slc32a1, Slc6a5, Pvalb, Calb1, Calb2*, CR, CB, PV). Calculation was performed as follow: (1) Relative to the total D2R-positive population:%HA = ((HA^+^ + Marker^+^) × 100)/HA^+^ indicating the proportion of D2R-expressing neurons co-expressing a given marker. (2) Relative to the total population of the marker of interests:%HA = ((HA^+^ + Marker^+^) × 100)/Marker^+^, indicating the proportion of marker-positive neurons also expressing D2R. For the iDISCO method, tissues were imaged by using an ultramicroscope Light Sheet (Miltenyi/LaVision BioTec) with the ImSpector Pro software (Miltenyi/LaVision BioTec). Three-dimensional images and movies were performed and treated with Imaris software (version 8.0.1, Oxford Instruments).

## Statistical analysis

Data were analyzed using Student *t*-test. Significance threshold was set at *p* < 0.05.

## Results

### Spatial distribution of D2R-expressing cells in the adult mouse spinal cord

**Figure 1:**
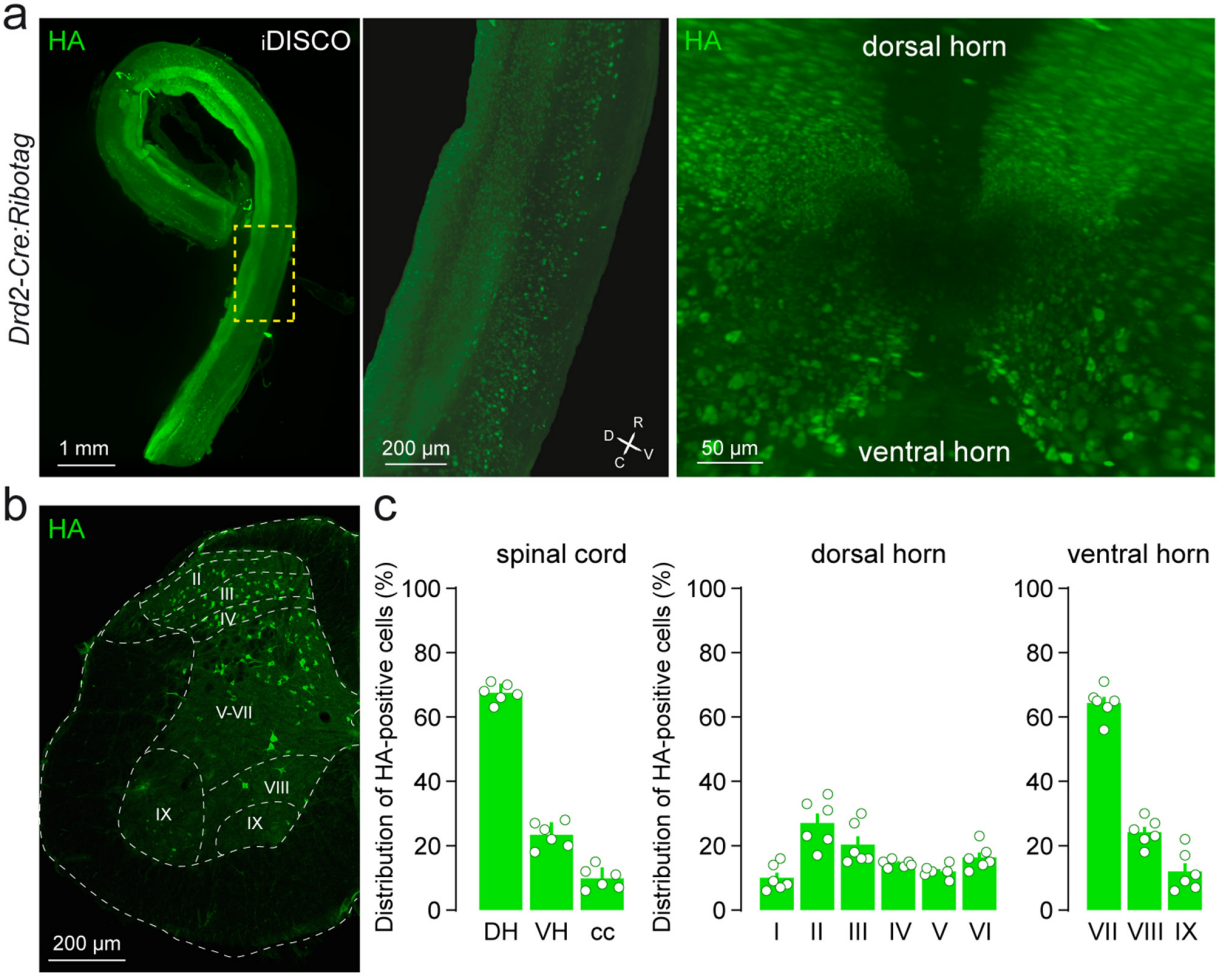
Whole-mounted imaging revealing the distribution of D2R cells in the spinal cord of *Drd2-Cre:Ribotag* mice. **(a)** From right to left: whole-mount view of the entire mouse spinal cord; a central panel presenting a zoomed-in view with the corresponding sagittal orientation of the spinal cord indicated; and a left panel showing a coronal view. HA-labeled cells are distributed throughout the spinal cord of adult *Drd2-Cre:Ribotag* mice. **(b)** Lumbar spinal cord coronal section from *Drd2-Cre:Ribotag* mice labeled with mouse anti-HA antibody (green). **(c)** Histograms showing the distribution of HA-labeled cells in the dorsal horn, ventral horn and the central canal as well as among the Rexed laminae delineation (3747 HA-counted cells; 2 sections per animal; *n* = 6 mice). HA, hemagglutinin; DH, dorsal horn; VH, ventral horn; cc, central canal.

**Figure 2:**
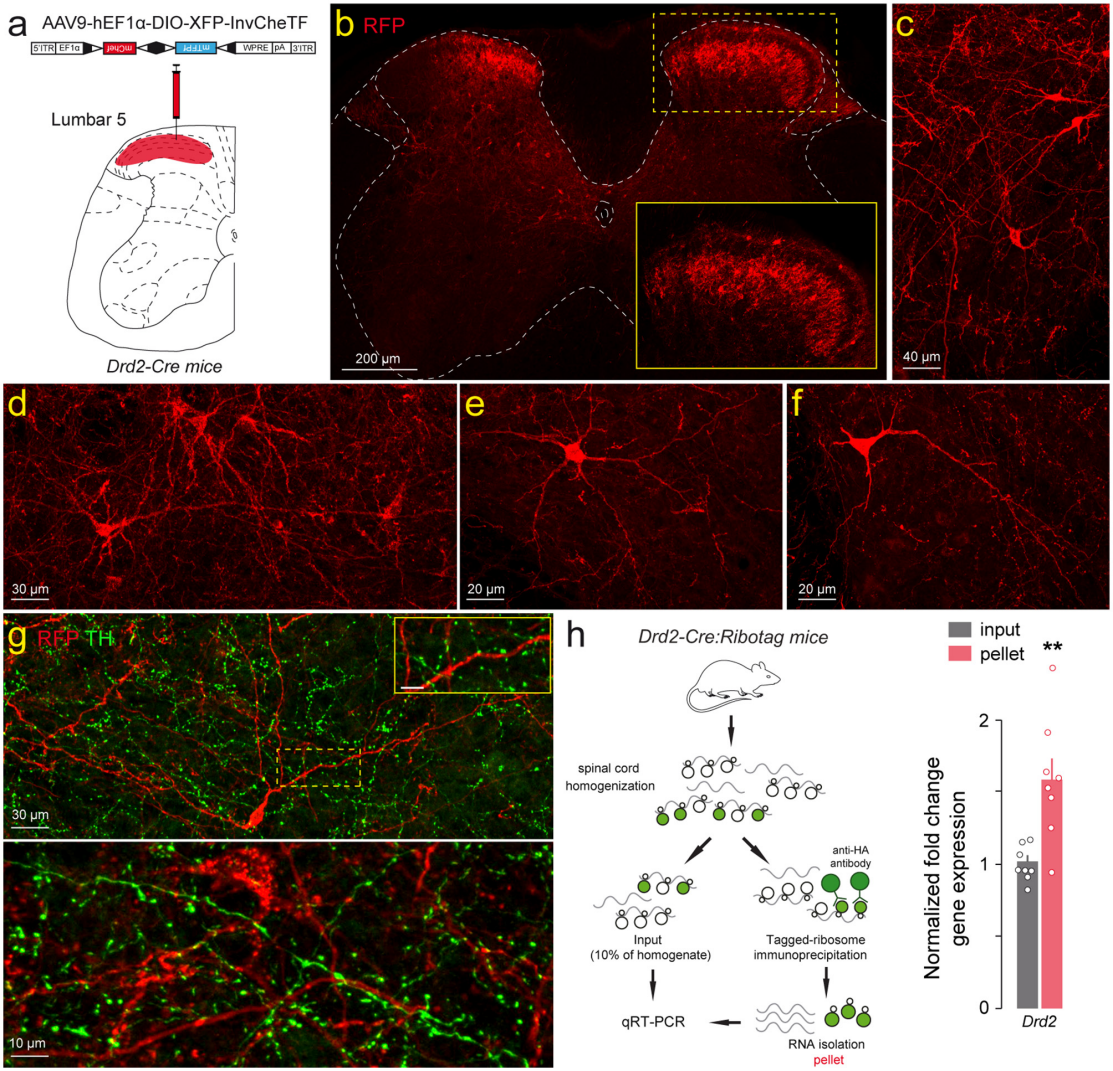
Lumbar spinal cord D2R cells are morphologically diverse and enriched in *Drd2* transcripts. **(a)** Schematic representation of the site for the Cre-dependent AAV9-hEF1^a^-DIO-XFP-InvCheTF injection into the dorsal horn of the lumbar spinal cord of adult *Drd2-Cre* mice. **(b, c)** Visualization of RFP-expressing neurons in the dorsal horn of the lumbar spinal cord. Insert in **b** is a high magnification of transduced cells in laminae II-III. **(d–f)** Example of isolated neurons expressing RFP under the promoter of D2R located in laminae V-VII. Note the morphological diversity of the transduced cells. **(g)** RFP (red) and TH (green) immunofluorescence in the lumbar spinal cord of *Drd2-Cre:Ribotag* mice showing TH-positive fibers surrounding D2R positive neurons located in laminae V (upper panel) and VI (lower panel). Insert is a high magnification image of area of the upper panel delineated by the yellow stippled rectangle showing TH-positive fibers that made appositions with nearby RFP-positive cells. **(h)** Cartoon illustrating the Ribotag methodology. qRT-PCR analysis of *Drd2* transcripts from pellet and input fractions of *Drd2-Cre:Ribotag* mice. *Drd2* gene product normalized to *Gapdh*. Data are presented as the fold change comparing the pellet fraction vs. the input (*n* = 8 pooled samples of 3 mice/pool). Mean ± SEM, Data analyzed by two-tailed Student's *t*-test. **p < 0.01. RFP, red fluorescent protein; TH, tyrosine hydroxylase.

The spatial distribution of SC D2R cells was analyzed using *Drd2-Cre:Ribotag* mice, which harbor the ribosomal protein Rpl22 tagged with the hemagglutinin (HA) epitope selectively in D2R-positive cells (Puighermanal et al., 2020, 2015). The visualization of HA-immunolabeling in cleared whole SC revealed that cells expressing D2R were present in all SC segments (cervical, thoracic, lumbar, sacral and coccygeal) and preferentially distributed in the dorsal horn (Figure 1a). We then focused our analysis on the lumbar SC since most anatomical and functional studies related to the actions of dopamine in the SC, have been carried out in this region (Sharples et al., 2020, 2014; Zhu et al., 2008). Quantitative analysis of HA-positive cells in lumbar SC slices confirmed their nonhomogeneous distribution, with D2R-expressing cells detected preferentially in the dorsal horn (67 ± 2.9%), followed by the ventral horn (23 ± 1.6%) and the central canal (10 ± 1.4%; Figures 1b, c). In the dorsal horn, laminae delineation based on Rexed's scheme revealed that HA-immunoreactive cells were present in all laminae (I-VI), with laminae II and III displaying the highest densities of HA-positive cells (27 ± 3.1% and 20 ± 2.7%, respectively; Figures 1b, c). Within the ventral horn, D2R-expressing cells were predominantly located in lamina VII (64 ± 2.1%) followed by lamina VIII (25 ± 1.7%) and lamina IX (11 ± 2.6%; Figure 1c). To confirm that the presence of HA-positive cells did not reflect residual developmental expression of the reporter gene, a Cre-dependent virus (AAV9-hEF1α-DIO-XFP-InvCheTF) was injected into the dorsal horn of the lumbar spinal cord of adult *Drd2-Cre* mice. This vector uses AAV9 to deliver a double-floxed inverted open reading frame driven by the constitutive hEF1α promoter, enabling expression of a fluorescent reporter (XFP) and a functional cassette (InvCheTF) exclusively in Cre-expressing cells. This design allows selective labeling of adult D2R-positive neurons while preventing baseline expression in Cre-negative cells (Figures 2a, b). Three weeks after injection, RFP-immunoreactivity was detected in heterogeneous cell types with morphologies that clearly indicated lumbar SC D2R cells were neurons (Figures 2c–f). Importantly, these D2R-positive cells were surrounded by a dense lattice of TH-positive fibers, as exemplified here in two sparse labeled cells located laminea V and VI (Figure 2g). Finally, the enrichment of *Drd2* transcript in the pellet fraction compared to the input fraction, following immunoprecipitation of HA-tagged ribosomes from SC extracts, confirmed both the specificity of the *Drd2-Cre:Ribotag* mice and the presence of *Drd2* gene products in the SC (Figure 2h).

### Identity of D2R-expressing cells in the adult mouse lumbar spinal cord

**Figure 3:**
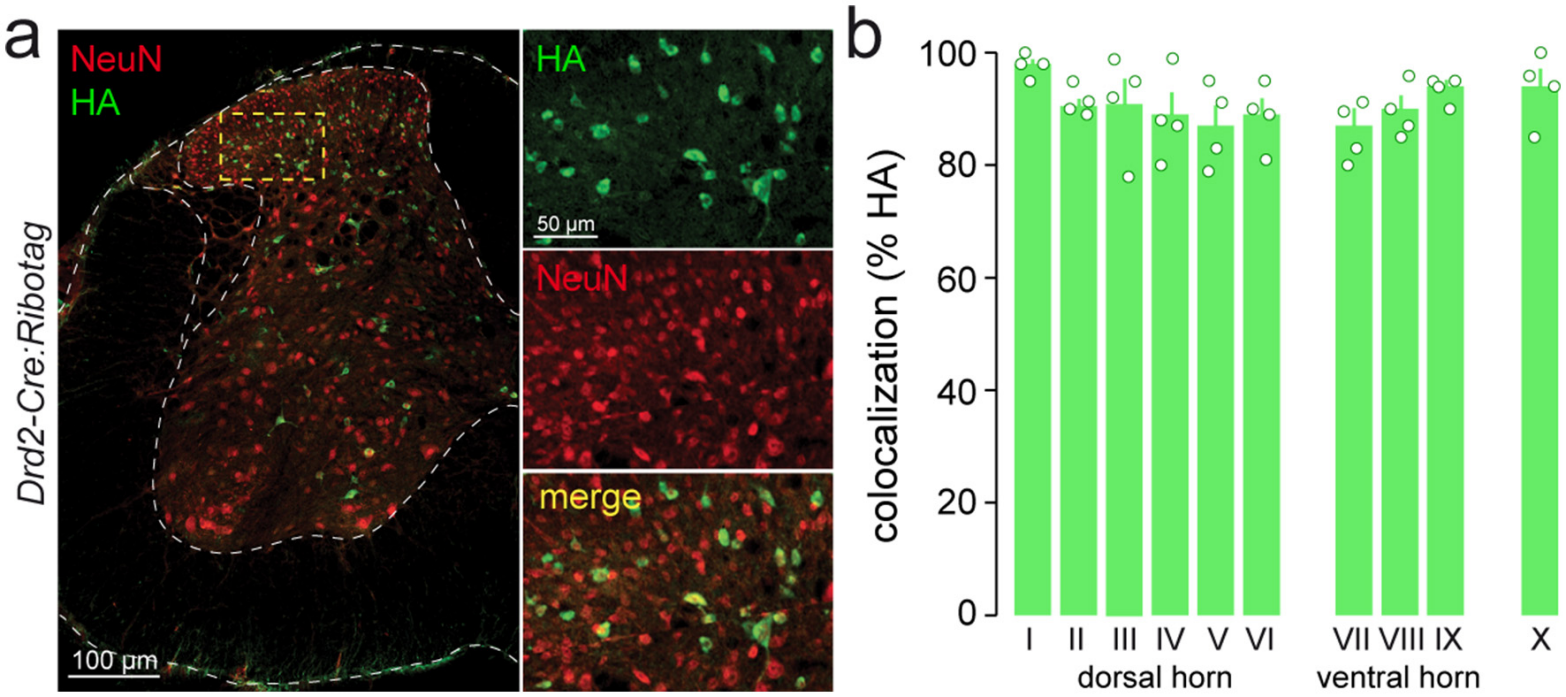
Lumbar spinal cord D2R-expressing cells are neurons. **(a)** Double immunofluorescence for HA (*green*) and NeuN (Fox-3, red) in the lumbar spinal cord of *Drd2-Cre:Ribotag* mice. Insert is a high magnification image of area delineated by the yellow stippled rectangle. **(b)** Histograms showing the co-expression of HA (green) and NeuN/Fox-3 (red) as a percentage of HA-positive cells in the Rexed laminae at the lumbar level of the spinal cord in the *Drd2-Cre:Ribotag* mice (1732 HA-counted cells; 2-3 sections per animal; *n* = 4 mice). HA, hemagglutinin.

**Figure 4:**
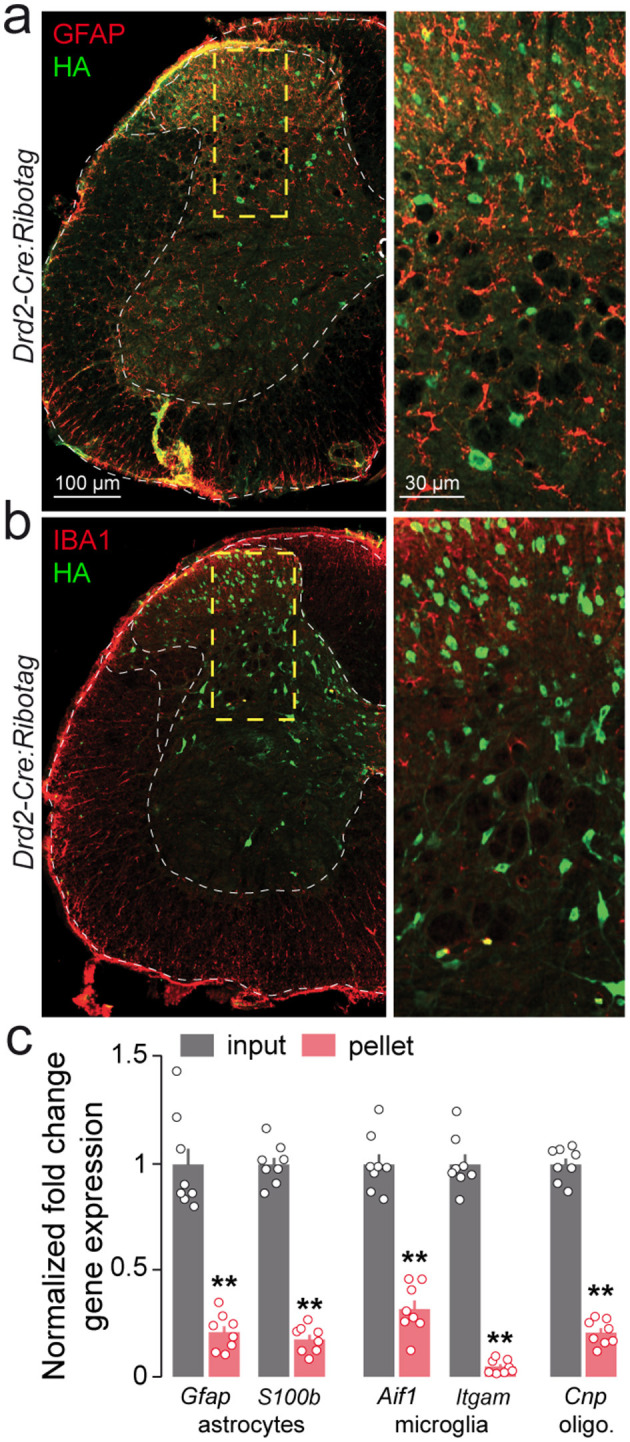
Lumbar spinal cord D2R cells are neither astrocytes nor oligodendrocytes or microglia. **(a**, **b)** Double immunofluorescence for HA (green) and GFAP (red) **(a)** or IBA1 (red,) **(b)** in the lumbar spinal cord of *Drd2-Cre:Ribotag* mice (*n* = 4 mice). High magnification images of areas delineated by the yellow stippled squares. No co-localization was found between HA cells and GFAP- or IBA1-immunoreactive cells. **(c)** qRT-PCR analysis showing the de-enrichment of markers of astrocytes (*Gfap, S100b*), microglia (*Aif1, Itgam*) and oligodendrocytes (*Cnp*) after HA-immunoprecipitation on spinal cord extract (pellet) compared to the input fraction (containing the mRNAs from all cellular types). Gene products normalized to *Gapdh*. Data are presented as the fold change comparing the pellet fraction vs. the input (*n* = 8 pooled samples of 3 mice/pool). Mean ± SEM, Data analyzed by two-tailed Student's *t*-test. **p < 0.01.

We next performed double immunostaining using neuronal and glial markers to further characterize the identity of lumbar SC D2R cells. We found that more than 90% of HA-immunoreactive cells co-expressed NeuN/Fox-3, a nuclear protein widely used as a neuronal marker (Kim et al., 2009; Figures 3a, b). In contrast, no HA-positive cells were co-labeled with GFAP or IBA1, suggesting that D2Rs were not expressed in astrocytes or microglia in the lumbar SC (Figures 4a, b). To further assess the cellular specificity of *Drd2* expression, we used the Ribotag approach, which isolates mRNAs actively undergoing translation in D2R-expressing cells, rather than measuring total transcript co-expression. Consistent with this interpretation, we observed a marked depletion of *Gfap* and *S100b* (astrocytic markers), *Aif1* and *Itgam* (microglial markers), and *Cnp* (oligodendrocytic marker) in the pellet fraction relative to the input. This selective reduction indicates that these glial transcripts are not being translated in D2R-positive cells under basal conditions (Figure 4c; Hernández-Ortega et al., 2024; Michetti et al., 2023; Yoon et al., 2017; Jurga et al., 2020; Mages et al., 2019). Altogether, these data indicate that D2Rs are expressed in neurons.

### Molecular identity of D2R-containing neurons in adult mouse lumbar spinal cord

Recent single-nucleus transcriptional profiling of the adult mouse SC revealed that lumbar SC neurons fall into three intermingled neuronal populations: cholinergic neurons, excitatory and inhibitory interneurons (Blum et al., 2021). We therefore examined the distribution of *Drd2* transcripts across these three neuronal populations.

**Figure 5:**
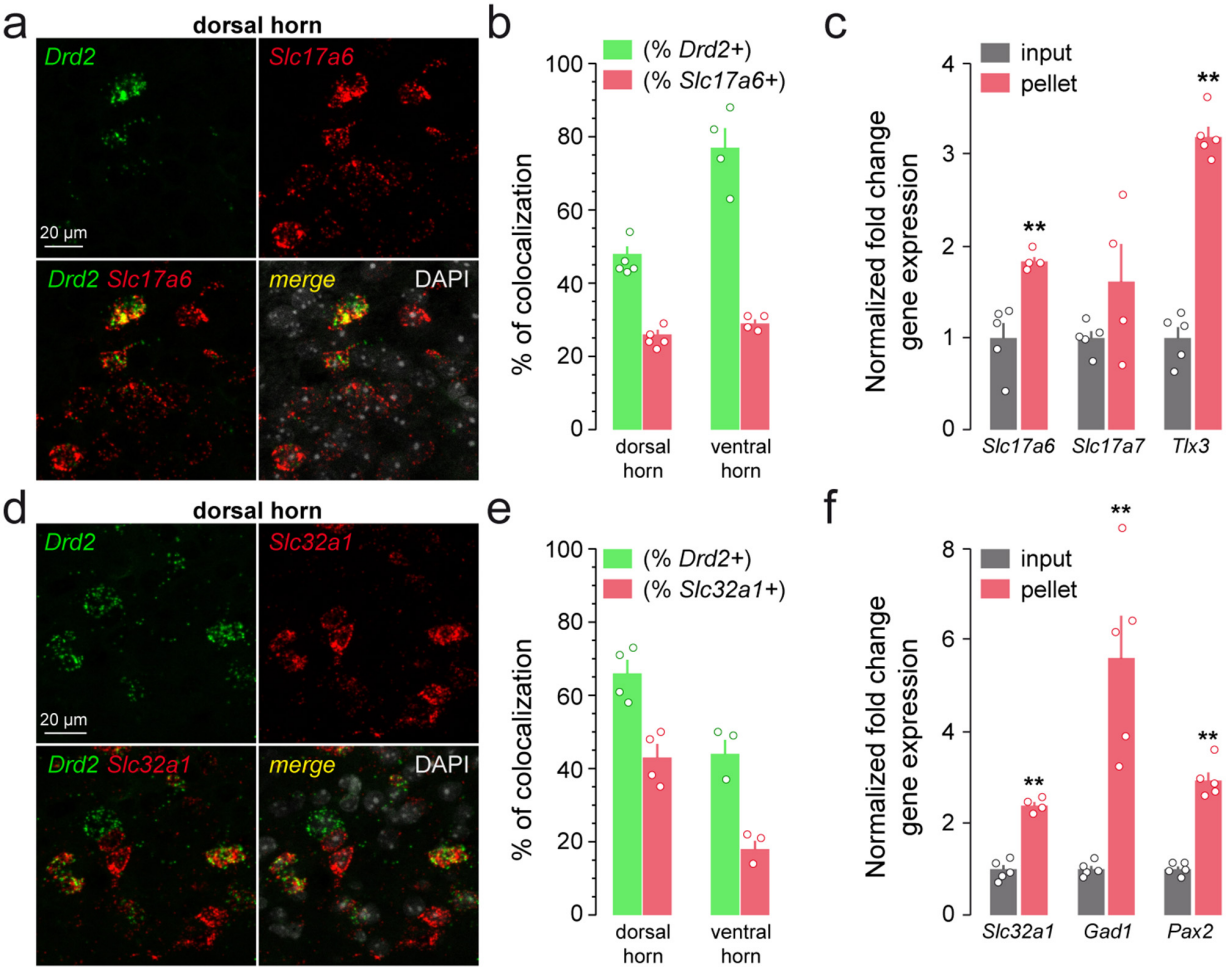
Distribution of *Drd2* among excitatory and inhibitory neurons. **(a** and **d)** Representative single scan confocal images of SC dorsal horn coronal sections from C57BL/6 mouse showing the distribution of *Drd2* (green) and *Slc17a6* (red) **(a)** or *Slc32a1* (red) **(d)** transcripts detected with single-molecular fluorescent *in situ* hybridization. Slides were counterstained with DAPI (white). **(b** and **e)** Histograms showing the co-expression as percentage of **(b)** *Drd2*-positive cells (green; 472 and 58 *Drd2*-positive cells counted in the dorsal and ventral horn respectively) or percentage of cells expressing *Slc17a6* (red; 874 and 149 *Slc17a6*-positive cells counted in the dorsal and ventral horn respectively; 5 slices per mouse, *n* = 5 mice in the dorsal horn and *n* = 4 mice in the ventral horn), and as percentage of **(e)** *Drd2*-positive cells (green; 431 and 80 *Drd2*-positive cells counted in the dorsal and ventral horn respectively) or percentage of cells expressing *Slc31a1* (red; 657 and 198 *Slc32a1*-positive cells counted in the dorsal and ventral horn respectively) (6 slices per mouse, *n* = 4 in the dorsal horn and *n* = 3 mice in the ventral horn). **(c** and **f)** qRT-PCR analysis showing the enrichment of markers of excitatory (*Slc17a6, Slc17a7, Tlx3*) (**c**) or inhibitory (*Slc32a1, Gad1, Pax2*) **(f)** neurons after HA-immunoprecipitation of spinal cord extract (pellet) compared to the input fraction. Gene products normalized to *Gapdh*. Data are presented as the fold change comparing the pellet fraction vs. the input (*n* = 5 pooled samples of 3 mice/pool). Mean ± SEM, Data analyzed by two-tailed Student's *t*-test. **p < 0.01.

Single-molecule fluorescent *in situ* hybridization analysis revealed that 48 ± 2.1% of *Drd2*-positive neurons in the dorsal horn and 77 ± 5.4% of those found in the ventral horn expressed transcripts encoding the vesicular glutamate transporter 2, *Slc17a6* (Figures 5a, b). Consistently, we found an enrichment of the actively translated transcripts *Slc17a6, Slc17a7*, and *Tlx3* in the pellet fraction relative to the input following HA-immunoprecipitation of spinal cord extracts from *Drd2-Cre:Ribotag* mice, confirming that *Drd2* gene products are present in excitatory neurons of the lumbar spinal cord (Figure 5c). Interestingly, the low percentage of HA/PKCγ-positive neurons indicated that D2R neurons located in laminae II and III of the dorsal horn do not belong to the class of PKCγ-positive excitatory interneurons (Polgár et al., 1999; Supplementary Figure 1).

**Figure 6:**
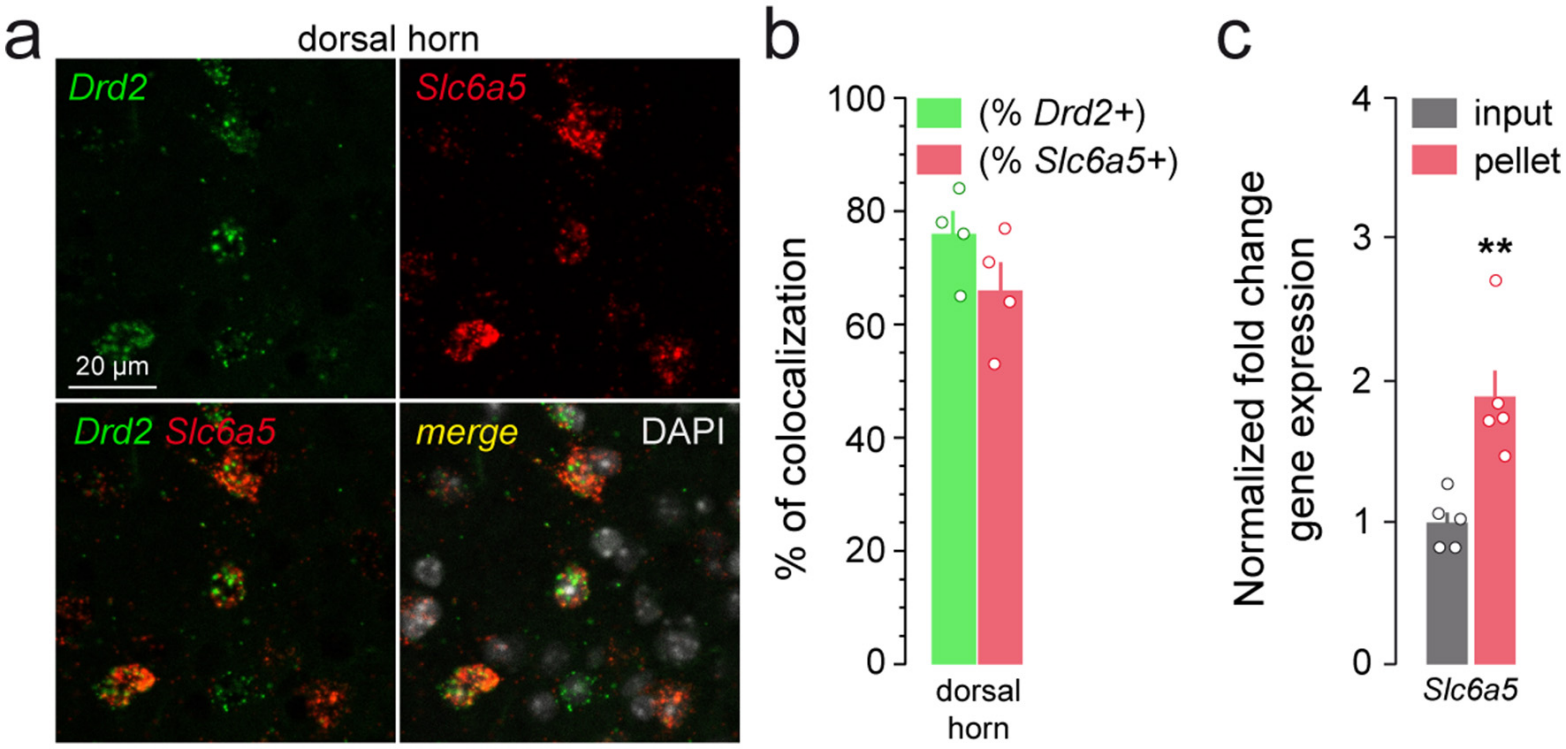
Distribution of *Drd2* among glycinergic neurons. **(a)** High magnification of confocal images of SC dorsal horn coronal sections from C57BL/6 mouse (*n* = 3 mice) showing the distribution of *Drd2* (green) and *Slc6a5* (red) expression detected with single-molecular fluorescent *in situ* hybridization. Slides were counterstained with DAPI (white). **(b)** Histograms showing the co-expression as percentage of *Drd2*-positive cells (green; 160 *Drd2*-positive cells counted in the dorsal) or percentage of cells expressing *Slc6a5* (red; 184 *Slc6a5*-positive cells counted in the dorsal) (3 slices per mouse, *n* = 4 mice). **(c)** qRT-PCR analysis showing the enrichment of glycinergic marker (*Slc6a5*) neurons after HA-immunoprecipitation of spinal cord extract (pellet) compared to the input fraction (containing the mRNAs from all cellular types). Gene product normalized to *Gapdh*. Data are presented as the fold change comparing the pellet fraction vs. the input (*n* = 5 pooled samples of 3 mice/pool). Mean ± SEM, Data analyzed by two-tailed Student's *t*-test. **p < 0.01.

Using similar approaches, we found that a fraction of *Drd2*-positive neurons expressed actively translated transcripts encoding for the vesicular inhibitory amino acid transporter (*Slc32a1*; Figures 5d, e) and the sodium- and chloride-dependent glycine neurotransmitter transporter (*Slc6a5*; Figures 6a, b). The presence of *Drd2* in both GABAergic and glycinergic neurons was further confirmed by the enrichment of *Gad1, Slc32a1, Pax2* and *Slc6a5* gene products after immunoprecipitation of HA-tagged ribosomes (Figures 5f, 6c). Finally, the lack of HA-labeled neurons co-expressing ChAT as well as the depletion of *Chat* and transcripts encoding the vesicular acetylcholine transporter (*Slc18a3*) indicated that motorneurons do not express D2R (Supplementary Figure 2). Altogether, our findings indicate that SC D2R neurons belong to both excitatory and inhibitory neuronal populations.

### Molecular heterogeneity of lumbar SC D2R neurons

**Figure 7:**
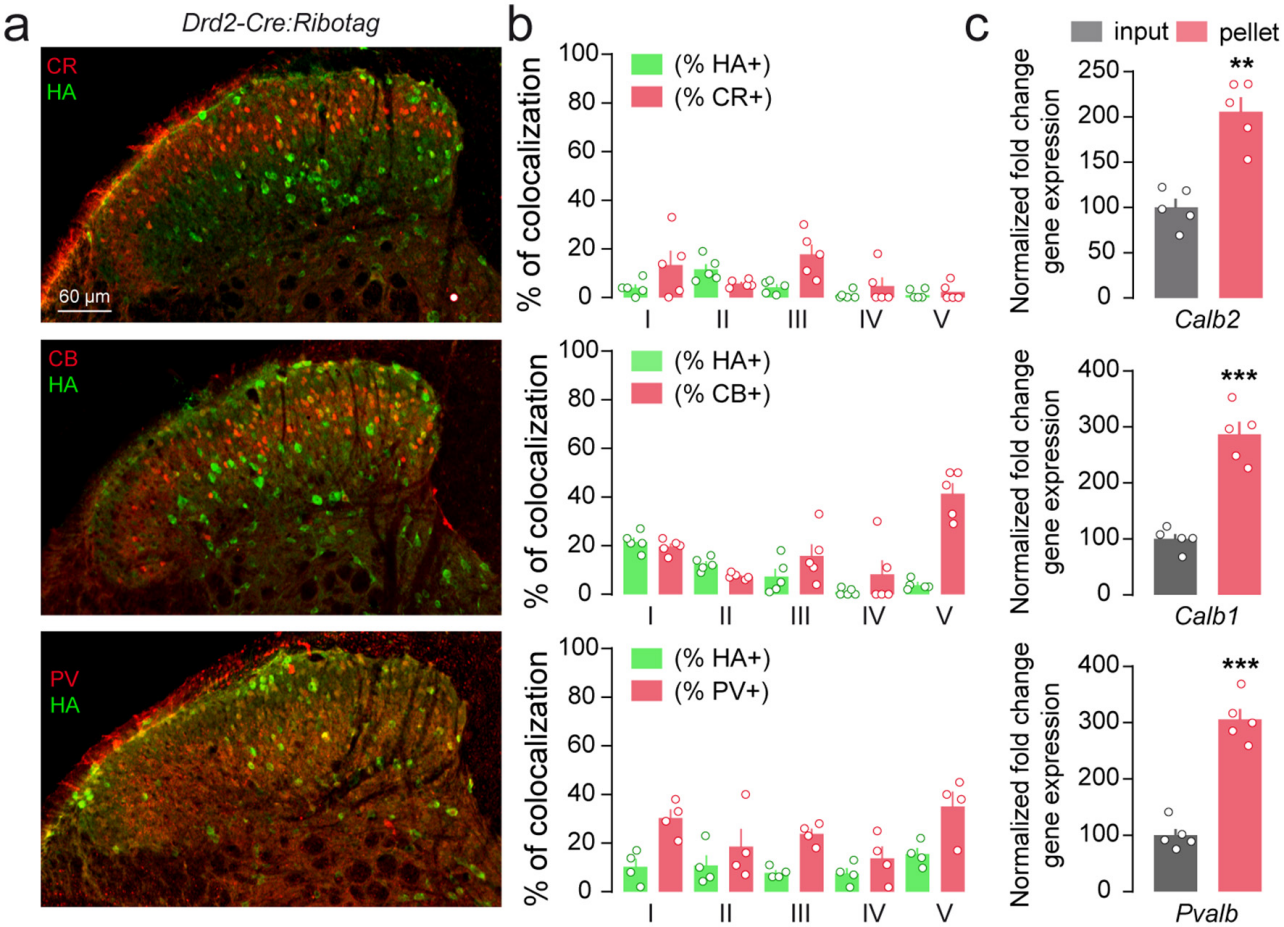
Distribution of lumbar spinal cord D2R cells among calcium-binding proteins. **(a)** Double immunofluorescence for HA (green) and calretinin (CR), calbindin-D28k (CB) and parvalbumin (PV) (red) in the dorsal horn of lumbar spinal cord of *Drd2-Cre:Ribotag* mice. **(b)** Histograms showing the co-expression of HA/CR, HA/CB and HA/PV as percentage of HA-positive cells (green, 807, 863 and 832 HA-labeled counted respectively) and as percentage of cells expressing calretinin (647 CR-labeled cells, 3 slices per mouse, *n* = 5 mice), calbindin-D28k (550 CB-positive cells, 3 slices per mouse, *n* = 5 mice) and parvalbumin (303 PV-positive cells, 3 slices per mouse, *n* = 4 mice) (red). **(c)** qRT-PCR analysis showing the enrichment of calretinin (*Calb2*), calbindin-D28k (*Calb1*) and parvalbumin (*Pvalb*) neurons after HA-immunoprecipitation of spinal cord extract (pellet) compared to the input fraction (containing the mRNAs from all cellular types). Gene product normalized to *Gapdh*. Data are presented as the fold change comparing the pellet fraction vs. the input (*n* = 5 pooled samples of 3 mice/pool). Mean ± SEM, Data analyzed by two-tailed Student's *t*-test. **p < 0.01, *** *p* < 0.001.

We next sought to determine whether lumbar SC D2R fall into molecularly distinct classes of excitatory and inhibitory neurons. Evaluation of the percentage of HA-positive neurons co-expressing the calcium-binding protein (CBPs), calretinin (CR), calbindin-D28k (CB) and parvalbumin (PV) indicated that lumbar SC D2R neurons seem to express the three CBPs (Figure 7). Among them, CR is the one displaying the lowest level of co-expression with D2R neurons (Figures 7a, b). HA-positive neurons co-expressing CB were preferentially found in laminae I (22 ± 1.8%) and II (13 ± 1.3%) while a fraction of the SC D2R neurons also expressed PV ranging from 5 ± 2.2% to 15 ± 2.5% depending on the laminae analyzed (Figures 7a, b). Despite the small number of samples in this histological approach, these results provide an initial indication suggesting heterogeneity within the D2R-expressing population. Similarly, transcripts encoding CR (*Calb2*), CB (*Calb1*) and PV (*Pvalb*) were enriched in the pellet fraction relative to the input following HA-immunoprecipitation of spinal cord extracts from *Drd2-Cre:Ribotag* mice (Figure 7c).

**Figure 8:**
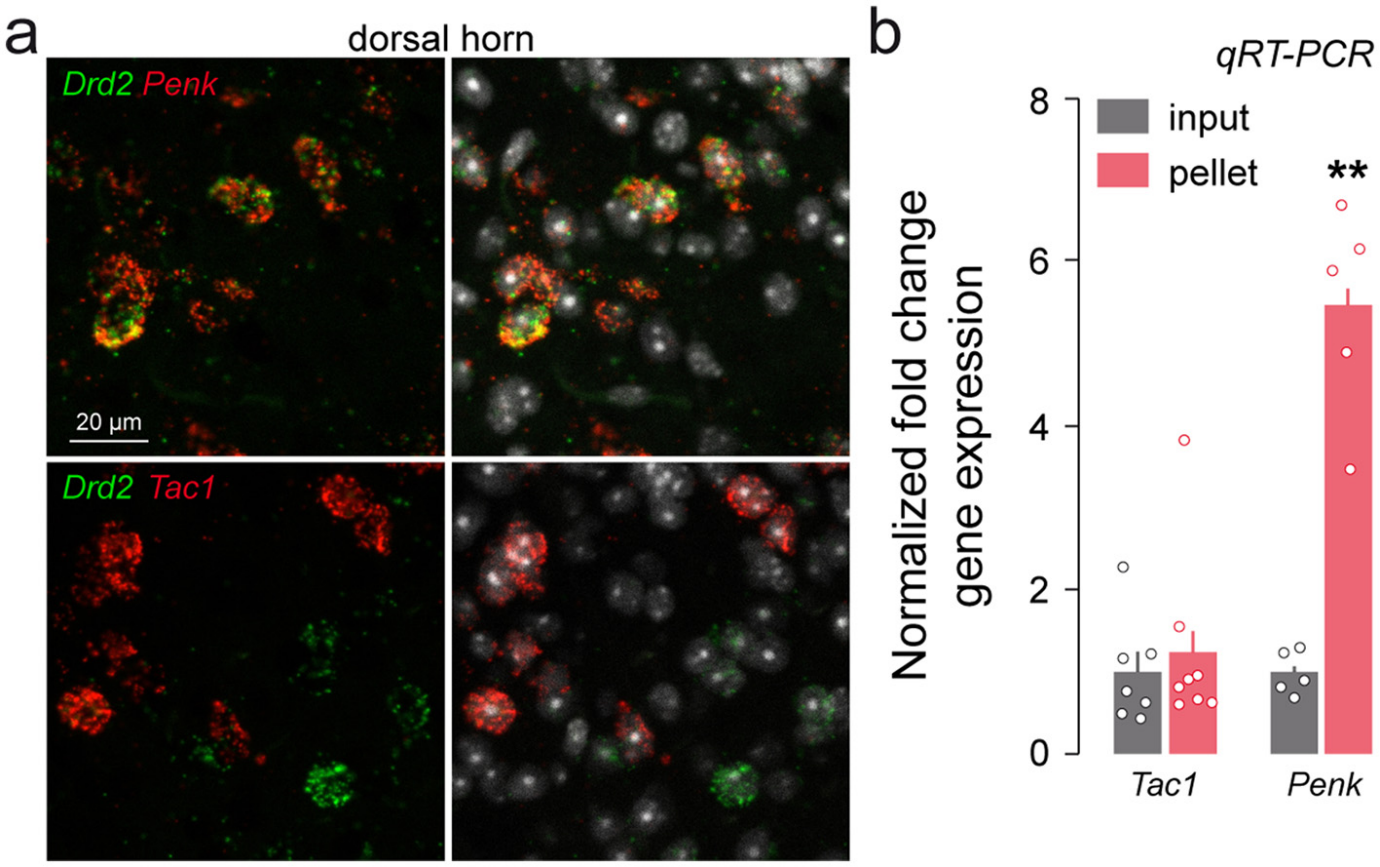
Distribution of *Drd2* among neuropeptides. **(a)** High magnification of confocal images of SC dorsal horn coronal sections from C57BL/6 mouse (*n* = 3 mice) showing the distribution of *Drd2* (green) and *Penk or Tac1* (red) expression detected with single-molecular fluorescent *in situ* hybridization. Slides were counterstained with DAPI (white). **(b)** qRT-PCR analysis showing the enrichment of *Penk* but not *Tac1* after HA-immunoprecipitation on spinal cord extract (pellet) compared to the input fraction (containing the mRNAs from all cellular types). Gene products normalized to *Gapdh*. Data are presented as the fold change comparing the pellet fraction vs. the input (*n* = 8 pooled samples of 3 mice/pool for *Tac1* and *n* = 5 pooled samples of 3 mice/pool for *Penk*). Mean ± SEM, Data analyzed by two-tailed Student's *t*-test. **p < 0.01.

Select combinations of neuropeptides and their receptors provide an additional level of classification for lumbar spinal neurons. Populations expressing both substance P (encoded by *Tac1*) and its receptor NK1R **(**encoded by *Tacr1*) represent distinct classes of excitatory and inhibitory interneurons involved in the transmission and modulation of nociceptive and sensory signals (Polgár et al., 2020; Shanley et al., 2011). In contrast, *Penk*-positive interneurons release enkephalin, an endogenous opioid peptide known to modulate pain processing (François et al., 2017; Fukushima et al., 2011). By combining multiplexed fluorescent *in situ* hybridization with transcript-level analysis, we found that lumbar SC D2R neurons were enriched in *Penk* transcripts (Figures 8a, b), whereas neither *Tac1* nor *Tacr1* were detected in *Drd2*-expressing neurons (Figures 8a, b and data not shown). Given the predominant localization of D2R neurons in the dorsal horn, a key site for pain integration, these co-expression patterns suggest that D2Rs may contribute to somatosensory and nociceptive modulation through molecularly distinct subsets of inhibitory and excitatory interneurons.

## Discussion

Over the past decade, significant progress has been made in the decoding the spatiomolecular identity of D2R-expressing neurons in the central nervous system. The use of *Drd2-Cre:Ribotag* mice has been instrumental in this quest, enabling the characterization of both the spatial distribution and the transcriptomic enrichment of D2R cells across multiple brain regions, including the dorsal and ventral striatum (Puighermanal et al., 2020), hippocampus (Puighermanal et al., 2015), cerebellum (Cutando et al., 2022) cerebral cortex (Cousineau et al., 2020; Khlghatyan et al., 2019) and more recently the external globus pallidus (Espallergues et al., 2024).

By employing a similar approach, we provide evidence that SC D2R cells are neurons, not glial cells, distributed throughout the rostrocaudal axis of the SC and preferentially localized to the laminae of the dorsal horn compared to the ventral horn. We also confirmed that D2Rs are expressed by spinal neurons (Zhao et al., 2007; Zhu et al., 2008) but not motorneurons, nor presynaptically on DA terminals projecting to the SC (Pappas et al., 2008). Together, these observations align with previous studies employing *in situ* hybridization, immunocytochemistry and autoradiography (Dubois et al., 1986; Kaur et al., 2014; Van Dijken et al., 1996; Yokoyama et al., 1994; Zhao et al., 2007; Zhu et al., 2008).

Our results, showing that D2Rs are expressed in distinct classes of inhibitory and excitatory interneurons, support prior evidence ascribing roles for SC D2Rs in diverse functions ranging from nociceptive modulation and autonomic functions to motor and sensorimotor integration (Asan et al., 2022; Clemens et al., 2012; Wang et al., 2021; Zhu et al., 2008). D2R modulation of spinal networks regulating analgesic responses is well-documented (Tang et al., 2021; Wang et al., 2021). Indeed, antinociceptive effects observed in response to cocaine administration (Kiritsy-Roy et al., 1994), intrathecal injection of DA (Liu et al., 1992) or direct stimulation of diencephalon-spinal DA neurons (Taniguchi et al., 2011) rely on spinal D2R activation. Aside from early work demonstrating that these antinociceptive effects depend on the ability of D2Rs to inhibit substantia gelatinosa neurons (Tamae et al., 2005; Taniguchi et al., 2011), little is known about the precise identity of SC D2R neurons mediating antinociception. Our study, revealing a high level of molecular heterogeneity of SC D2R neurons across inhibitory and excitatory interneuron populations might provide important clues. Indeed, it is tempting to speculate that subsets of parvalbumin-positive interneurons and/or excitatory PKCγ-positive interneurons in the superficial laminae that express D2R could contribute to this regulation, as both cell types integrate nociceptive inputs from C-fibers (Booker and Wyllie, 2017; Gradwell et al., 2022; Neumann et al., 2008).

Interestingly, evidence indicates that D2R-mediated antinociception displays some specificity with respect to the nature of the stimuli. For example, intrathecal injection of the D2R agonist quinpirole produces antinociceptive effects against mechanical, but not thermal stimuli, a phenomenon thought to result from the direct inhibition of nonpeptidergic dorsal root ganglion neurons innervating the dorsal horn of the SC (Almanza et al., 2019). Indirect modulation of sensory afferent transmission may also contribute, as D2R activation has been shown to reduce primary afferent depolarization evoked by stimulation of low-threshold dorsal roof afferents (Milla-Cruz et al., 2020), as well as depress C-fiber-evoked field potentials in the spinal dorsal horn (Aira et al., 2014; Lapirot et al., 2011). Interestingly, our analysis further suggests that additional mechanisms could be involved. Notably, D2Rs appear to be highly enriched in dorsal horn sensory neurons expressing enkephalin identified as key integrators of nociceptive signals (Bai et al., 2020; François et al., 2017; Fukushima et al., 2011). We therefore hypothesize that D2R-mediated antinociception of mechanonociceptors may occur, at least in part, through actions on enkephalinergic neurons known to modulate mechanical pain (François et al., 2017).

Descending catecholaminergic systems also contribute jointly, but differentially, to various aspects of locomotor control (Sharples et al., 2014). While the initiation of locomotion seems to primarily rely on the noradrenergic system (Rossignol et al., 1998), locomotion-induced spinal DA release is thought to play a more prominent role in modulating the locomotor pattern (Barbeau and Rossignol, 1991; Gerin et al., 1995; Gerin and Privat, 1998). Supporting this hypothesis, DA has been shown to induce slow locomotor-like rhythmic activity in isolated SC preparations, a process partly dependent on spinal D2R-mediated signaling (Barrière et al., 2004; Gordon and Whelan, 2006; Sharples et al., 2020). This regulation is unlikely to occur directly at the level of motorneurons as neither our study nor previous reports detected *Drd2* transcripts in this neuronal population (Van Dijken et al., 1996). Consistently, DA-mediated modulation of AMPA currents in spinal motorneurons has been shown to be independent of D2R activation (Han and Whelan, 2009). Instead, indirect regulation is likely, as D2R activation suppresses recurrent excitatory feedback projecting onto rhythm-generating circuitry (Humphreys and Whelan, 2012; Maitra et al., 1993).

Finally, early evidence establish a role of D2R-mediated signaling in the regulation of the Straub tail reaction (Zarrindast et al., 1993; Hasegawa et al., 1990). Indeed, Straub tail reaction induced by morphine was reduced by prior administration of the D2R antagonists (Hasegawa et al., 1990). Importantly, systemic or intrathecal injection of the D1R/D2R agonist apomorphine was sufficient to cause Straub tail reaction which was dependent on D2R-mediatied signaling (Zarrindast et al., 1993; Hasegawa et al., 1990). Although the exact mechanism remains to be established, our anatomical study supports the hypothesis that such regulation could involve D2R neurons located in the SC. Future studies will be necessary to precisely determine which SC D2R neurons identified in the present study contributes to the Straub tail reaction induced by morphine or apomorphine.

By providing a comprehensive anatomical and molecular mapping of spinal D2R neurons, our study helps to fill important gaps in our understanding of D2R signaling modulates spinal circuits. Notably, future studies will be required to determine whether distinct D2R signaling mechanisms operate within the heterogeneous population of spinal D2R neurons, as previously demonstrated in the cerebral cortex (Cousineau et al., 2020; Tseng and O'Donnell, 2004) and the hippocampus (Etter and Krezel, 2014; Tuduri et al., 2025).

## Data Availability

The raw data supporting the conclusions of this article will be made available by the authors, without undue reservation.
