# Supplementary material for: Mouse spinal cord cellular mapping of dopamine D2 receptors-containing cells
Source: Front Neuroanat. 2026 Jan 20;19:1724268. doi: 10.3389/fnana.2025.1724268 (PMC12864406; doi:10.3389/fnana.2025.1724268)
Supplement: Supplementary file 1 [file Data_Sheet_1.pdf]

Mouse spinal cord cellular mapping of dopamine D2 receptors-containing cells

Pauline Tarot[1], Laura Cutando[1*], Laia Castell[1#], Emma Puighermanal**[1*],** Emmanuel Valjent[1, 2]

[1] IGF, University Montpellier, CNRS, Inserm, Montpellier, France

[2] INM, University Montpellier, Inserm, Montpellier, France

Present address

[*]Institute of Neuroscience, Universitat Autònoma de Barcelona, Bellaterra, Spain. Departament de Biologia Cellular, Fisiologia i Immunologia, Universitat Autònoma de Barcelona, Barcelona, Spain.

[#] Department of Neuroscience, Northwestern University Feinberg School of Medicine, Chicago, IL 60611, USA

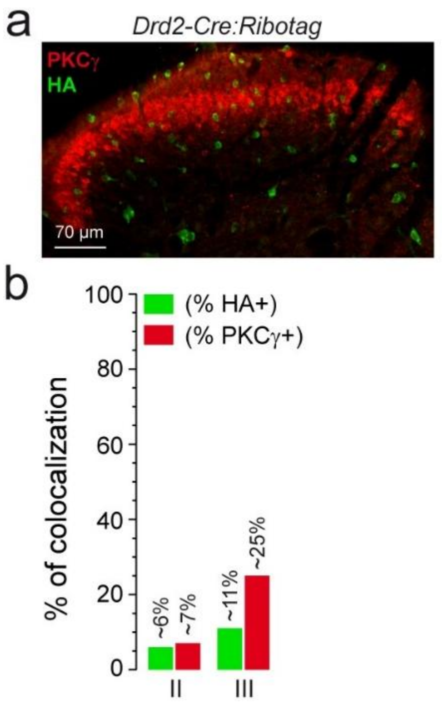

Supplemental Figure 1. Distribution of lumbar spinal cord D2R cells among PKCγ. (**a**) Double immunofluorescence for HA (green) and PKCγ (red) in the dorsal horn of lumbar spinal cord of *Drd2-Cre:Ribotag* mice. (**b**) Histograms showing the co-expression of HA/ PKCγ as percentage of HA-positive cells (green, 142 and 105 HA-labeled counted in laminae II and III, respectively) and as percentage of cells expressing PKCγ (red, 128 and 48 PKCγ-labeled counted in laminae II and III, respectively) (6 slices per mouse, n = 2 mice).

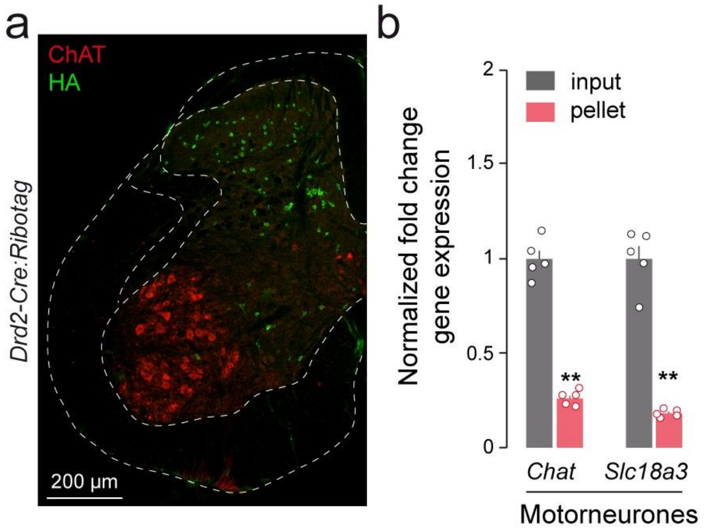

Supplemental Figure 2. Lumbar spinal cord *Drd2* transcripts are not present in motorneurons. (**a**) Double immunofluorescence for HA (green) and cholineacetyltransferase (ChAT) (red) in the ventral horn of lumbar spinal cord of *Drd2-Cre:Ribotag* mice. (**b**) qRT-PCR analysis showing the de-enrichment of motorneurones markers (*Chat*, *Slc18a3*) neurons after HA-immunoprecipitation on spinal cord extract (pellet) compared to the input fraction (containing the mRNAs from all cellular types). Gene product normalized to *Gapdh*. Data are presented as the fold change comparing the pellet fraction versus the input (n = 5 pooled samples of 8 mice / pool). Data are presented as Mean ± SEM and analyzed by two-tailed Student's *t*-test. $^{**}p < 0.01$.

Supplemental Table 1: List of Primary antibodies

Antibodies	Species	Dilution	Supplier	References
HA	Mouse	1:1000	Biolegend	#clone 16B12
HA	Rabbit	1:500	Rockland	#600-401-384
Parvalbumin	Mouse	1:500	Millipore	#MAB1572
Parvalbumin	Rabbit	1:1000	Swant	#PV 25
Calbindin-D28k	Rabbit	1:1000	Swant	#CB382
Calretinin	Mouse	1:500	Swant	#6B3
Calretinin	Rabbit	1:1000	Swant	#7699/3H
GFP	Chicken	1:1000	Invitrogen	#A10262
RFP	Rabbit	1:1000	MBL	#PM005
ChAT	Rabbit	1:1000	Millipore	#AB143
NeuN	Mouse	1:500	Millipore	#MAB377
IBA1	Rabbit	1:500	Wako	#019-19741
GFAP	Rabbit	1:1000	Dako	#N1506
TH	Rabbit	1:500	Millipore	#AB152
PKCγ	Guinea Pig	1:1000	Frontier Institut	#GP-Af350

Supplemental Table 2: List of Secondary antibodies

Antibodies	Species	Dilution	Supplier	References
Anti-mouse A488	Goat	1:500	Invitrogen	A11001
Anti-Rabbit CY3	Goat	1:500	Jackson immunoresearch	115-165-075
Anti-Goat CY3	Goat	1:500	Invitrogen	A10520
Anti-Rabbit A488	Goat	1:500	Invitrogen	A11034
Anti-Guinea Pig A647	Goat	1:500	Invitrogen	A21450
Anti-Chicken A488	Goat	1:500	Invitrogen	A11039

Supplemental Table 3: List of Probes RNAscope

**Probes (gene)**	**Probes (Protein)**	**Supplier**	**References**
Drd2	D2R	ACDBio	406501-C3
Slc32a1	VGAT	ACDBio	319191-C2
Slc17a6	VGLUT2	ACDBio	409741-C1
Slc17a7	VGLUT1	ACDBio	416631-C1
Penk	ENKEPHALINE	ACDBio	318761-C1
Slc6a5	GLYT2	ACDBio	409741-C1
Tac1	SUBSTANCE P	ACDBio	410351-C1

Supplemental Table 4: List of primers

Genes	Proteins	Primers sequences
β-actin	β-Actin	**F**: CCCCCAGCCAAGAAAGCTAT<br>**R**: GCCCCACCGTGTGACATC
Gapdh	GAPDH	**F**: CAACTACATGGTCTACATGTTCCAA<br>**R**: CCCATTCTCGGCCTTCACT
Slc32a1	VGAT	**F**: TCACGACAAACCCAAGATCAC<br>**R**: GTCTTCGTTCTCCTCGTACAG
Gad1	GAD	**F**: TTGTGCTTTGCTGTGTTTTAGAGA<br>**R**: CCCCCTGCCCAAAGATAGAC
Slc18a3	VACHT	**F**: GGCCTCGCTACCCCACAGAA<br>**R**: CCCAGGCCAATAAGCAGCGG
Slc17a6	VGLUT2	**F**: CGCTGTCGGGGATGGTTTGC<br>**R**: GTGGACGAGTGCAGCAATGAGG
Penk	ENKEPHALINE	**F**: CTACAGTGCAGGCGGAATGC<br>**R**: GTCCTTCACATTCCAGTGTGC
Chat	CHAT	**F**: CCATTGTGAAGCGGTTTGGG<br>**R**: GCCAGGCGGTTGTTTAGATACA
Drd2	D2R	**F**: CTCTTTGGACTCAACAACACAGA<br>**R**: AAGGGCACGTAGAACGAGAC
Cnp	CNP	**F**: GCTGCACTGTACAACCAAATTCTG<br>**R**: ACCTCCTGCTGGGCGTATT
Gfap	GFAP	**F**: AGCGAGCGTGCAGAGATGA<br>**R**: AGGAAGCGGACCTTCTCGAT
Aif1	IBA1	**F**: CCCCCAGCCAAGAAAGCTAT<br>**R**: GCCCCACCGTGTGACATC
Itgam	CD111-B	**F**: ATGGTCACCTCCTGCTTGTGAG<br>**R**: CCAGCAGTGATGAGAGCCAAGA
S100b	S-100B	**F**: TTCCACCAGTACTCCGGGCG<br>**R**: GCGACGAAGGCCATGAACTCC
Tlx3	TLX3	**F**: AAATGACGGACGCGCAGGTC<br>**R**: GAAGGCGTCGTGTTGCAGCT
Pax2	PAX2	**F**: CCATCCCCAGTACACCGCCT<br>**R**: TCCCCGCGGTAACTAGTGGC
Tac1	SUBSTANCE P	**F**: TGGACTAATGGGCAAAAGAGC<br>**R**: CGTTCACTGCTCACTGACAC
Slc17a7	VGLUT1	**F**: TGTGCCCCATCATCGTGGGT<br>**R**: CGCTCATCTCCTCCGGCTCT
Slc6a5	GLYT2	**F**: TGATGCTCGCCTGCTCCGTT<br>**R**: CCCGCGGTGCTGAGCTAAGA
